# Functional Role of PilA in Iron Acquisition in the Cyanobacterium *Synechocystis* sp. PCC 6803

**DOI:** 10.1371/journal.pone.0105761

**Published:** 2014-08-26

**Authors:** Jacob J. Lamb, Ryan E. Hill, Julian J. Eaton-Rye, Martin F. Hohmann-Marriott

**Affiliations:** 1 Department of Biotechnology, PhotoSynLab, Norwegian University of Science and Technology, Trondheim, Norway; 2 Department of Biochemistry, University of Otago, Dunedin, New Zealand; University of Freiburg, Germany

## Abstract

Cyanobacteria require large quantities of iron to maintain their photosynthetic machinery; however, in most environments iron is present in the form of insoluble iron oxides. Whether cyanobacteria can utilize these sources of iron, and the potential molecular mechanisms involved remains to be defined. There is increasing evidence that pili can facilitate electron donation to extracellular electron acceptors, like iron oxides in non-photosynthetic bacteria. In these organisms, the donation of electrons to iron oxides is thought to be crucial for maintaining respiration in the absence of oxygen. Our study investigates if PilA1 (major pilin protein) may also provide a mechanism to convert insoluble ferric iron into soluble ferrous iron. Growth experiments supported by spectroscopic data of a strain deficient in *pilA1* indicate that the presence of the *pilA1* gene enhances the ability to grow on iron oxides. These observations suggest a novel function of PilA1 in cyanobacterial iron acquisition.

## Introduction

In the oxidative environment of Earth, organisms must contend with the problem of accessing essential elements, which are locked into insoluble oxides. In particular, the bioavailability of iron is limited due to the tendency of Fe^3+^ to form insoluble minerals (i.e. goethite, hematite) [1].

### Iron acquisition in bacteria

Given the limited bioavailability of iron, several acquisition strategies have evolved in bacteria. Siderophore-mediated iron uptake is one such strategy, involving the synthesis and secretion of low-molecular-weight iron chelators that tightly bind Fe^3+^. This results in a ferrisiderophore complex that is transported as a whole into the cell where the iron can then be removed for cellular utilization. This form of siderophore-mediated iron uptake has been studied extensively in Gram-negative bacteria such as *Escherichia coli* and *Pseudomonas aeruginosa* [2], [3], [4], [5]. Citrate also possesses Fe^3+^ chelating characteristics and many bacteria possess a citrate-mediated mechanism for iron uptake. The uptake of heme through hemophores (proteins that are synthesized and excreted) is another iron-acquisition strategy that has only been found in Gram-negative bacteria [6], [7].

Recently, the Type IV pili of non-photosynthetic soil bacteria have been shown to provide another strategy for iron acquisition. Observations using Scanning Tunneling Microscopy (STM) techniques demonstrated that these thick pili structures are electrically conductive, coining the name ‘bacterial nanowires’ [8], [9]. These pili are composed of pilins that form extracellular protein fibers and facilitate electron donation to external electron acceptors like metal oxides, thus facilitating respiration in anaerobic conditions [8], [9]. In addition to sustaining respiration, another - so far untested - consequence of reducing metal oxides is that this may also confer the ability to unlock iron that is essential for bacterial growth.

### Iron utilization in cyanobacteria

Cyanobacteria employ a variety of strategies to access iron from the environment. Although siderophore production has been observed in several cyanobacteria species [10], detailed analysis has shown that genes for siderophores are not present in many cyanobacteria species, including *Synechocystis* sp. PCC 6803 [11]. Despite this, it has been suggested that *Synechocystis* sp. PCC 6803 can obtain iron bound to exogenous siderophores [12], [13] that are produced by other organisms in proximity. Citrate has the ability to chelate ferric iron, and ferric dicitrate uptake systems are present in the genomes of many cyanobacteria, including *Synechocystis* sp. PCC 6803. Alternatively, an iron uptake pathway for cyanobacteria involving a reductive step has been evaluated in recent studies, suggesting extracellular or periplasmic reduction occurs followed by transport of the iron through the plasma membrane into the cell [14].

### Iron utilization in *Synechocystis* sp. PCC 6803

Several mechanisms for iron uptake are well-understood in *Synechocystis* sp. PCC 6803. In the plasma membrane of *Synechocystis* sp. PCC 6803 there are two transporters FutABC and FeoB, suggested to transport free Fe^3+^ and Fe^2+^, respectively [12], [13], [15]. A ferric dicitrate uptake system is coded by the genes *slr1318–1319 and slr1491–1492* in *Synechocystis* sp. PCC 6803. Ferric ammonium citrate has been used as an iron source in cyanobacterial growth media, including BG-11 for *Synechocystis* sp. PCC 6803 [16].

Kranzler et al. [14] have presented data suggesting that *Synechocystis* sp. PCC 6803 is capable of acquiring iron through reduction of Fe^3+^ substrates before transport through the plasma membrane. Although the location of iron reduction is yet to be defined, it is proposed to be outside of the cell, on the surface of the outer membrane or in the periplasmic space [14]. This reductive two-step model for iron uptake allows for the utilization of a variety of inorganic and organic iron sources, thereby eliminating the presence for specific ferrisiderophore transporters. Following reduction, iron may be transported as Fe^2+^ or, in some cases, reoxidized to Fe^3+^ and then transported [17], [18], [19] across the cytoplasmic membrane. This ‘reductive activation’ may provide a mechanism for accessing the great variety of ferric iron conjugates and iron chelators cyanobacteria may encounter in their natural environment.

### Iron-stress-induced chlorophyll-binding protein regulation

Iron limitation induces a remarkable remodeling of the photosynthetic machinery in cyanobacteria. Under iron stress IsiA (an iron-starvation-induced chlorophyll-binding protein), is expressed. IsiA can form ring structures around Photosystem I (PSI) [20], [21] and binds up to 50% of cellular chlorophyll [22]. Accumulation of IsiA leads to spectral changes that can be readily observed at room temperature and 77 K. The transcription of *isiA* in *Synechocystis* sp. PCC 6803 is regulated by transcriptional activation of the corresponding bi-cistronic *isiAB* operon [23] that is controlled by the ferric uptake regulator protein (Fur) [24]. Another regulation of the *isiAB*-operon involves an internal asRNA, *isiR* [25], [26]. A strain, in which the *isiA* gene has been inactivated, shows a transcriptional up-regulation of several pilin genes [27]. This observation indicates that the expression of pilins interfaces with a regulatory network that controls the expression of IsiA in response to stress conditions, including iron depletion.

### Pilins in *Synechocystis* sp. PCC 6803

In *Synechocystis* sp. PCC 6803, functional pili are essential for twitching motility and DNA uptake [28], [29], [30], [31]. The genome of *Synechocystis* sp. PCC 6803 contains a large number of genes that show clear pilin characteristics. Four pilin genes are encoded individually (*slr0079, slr1120, slr1456* and *sll1359*), but there are also three operons in which pilins are arranged in consecutive order. One operon consists of the genes *sll1693–1696*, where the protein encoded by *sll1694* (the *pilA1* gene) is the main constituent of the extracellular pili [28], [29], [30], [31]. Deletion of this pilin results in an inability to take up external DNA, impairment of mobility, and the absence of pili as shown by negatively stained electron micrographs [31]. The protein encoded by *sll1695* (the *pilA2* gene) is another pilin that has been suggested to be localized on the cytoplasmic surface of the cytoplasmic membrane [29], [30]. The function of proteins encoded by *sll1693* and *sll1696* are not known. The two additional operons containing pilin genes are *slr1928–1931* and *slr2015–2017*. Unlike the pilins encoded by the operon *slr1928–1931* [31], the pilins encoded within and the other operon (*slr2015* and *slr2016*) are crucial for locomotion [29].

### Aim of this study

There are several lines of evidence suggesting a role of pili in mediating the reduction of iron oxides in bacteria [8], [9], and reduction of iron has been implicated in making iron available for cyanobacteria [14]. As iron is an essential element and induces a strong phenotypic response in cyanobacteria, we decided to assess iron bioavailability as a function of the presence of PilA1 in *Synechocystis* sp. PCC 6803.

## Results

### Δsll1694 strain construction

The PilA pilin encoded by *sll1694* is the main component of Type IV pili [29], [31]. A mutant of *Synechocystis* sp. PCC 6803 was generated in which *sll1694* was replaced by a kanamycin-resistance cassette (Fig. S1 in File S1). Mutant segregation, i.e. the replacement of *sll1694* in all copies of chromosomes in a *Synechocystis* sp. PCC 6803 strain, was achieved by plating out *Synechocystis* sp. PCC 6803 cells that were transformed with the Δ*sll1694* deletion plasmid on BG-11 media, supplemented with the antibiotic kanamycin. Complete segregation was confirmed by colony PCR (Fig. S2 in File S1).

### Characterization of pilA1 deletion strain

Liquid cultures of wild type, and the Δ*sll1694* strain were grown in liquid medium in order to assess the presence of PilA1 in these two strains. Once cultures reached an OD of 0.8, they were harvested and PilA were sheared off the cells by vortexing [32]. The concentrated supernatant was analyzed by SDS-PAGE (Fig. S3 in File S1). The main band visible in wild type (located at ∼20 kDa), was excised and characterized by mass spectrometry. Analysis of the sample protein resulted in the sequence of a 13 amino acid fragment (SMSGGTFYDSGTR). This sequence matched to the *sll1694* gene product (PilA1), confirming that the gene product of *sll1694* is the main constituent of the cell supernatant [28], [29], [30], [31] and the Δ*sll1694* strain had been inactivated.

### Growth analysis

**Figure 1:**
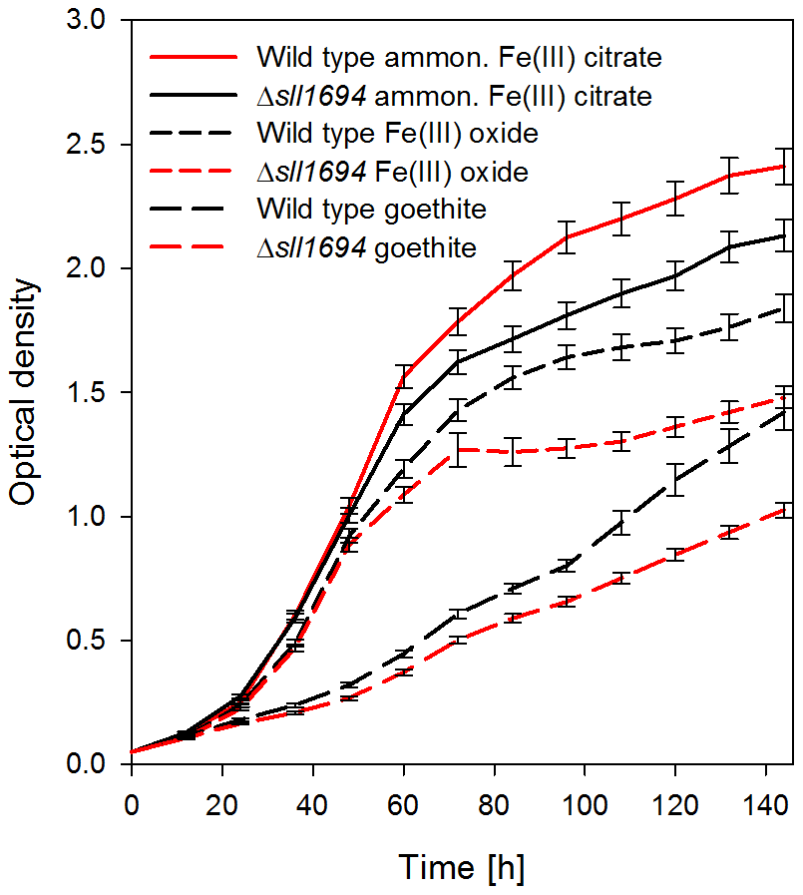
Liquid growth characteristics. Photoautotrophic growth characteristics of wild type and the Δ*sll1694* strain in liquid BG-11 with ammonium iron(III) citrate, iron(III) oxide, or goethite as the exclusive iron source. Trend shown is the average of three separate experiments. Error bars indicate the standard error of the three experiments.

The growth characteristics of wild type and the Δ*sll1694* strain in liquid BG-11 medium (without added glucose or atrazine) on different iron sources were characterized. Cells grew fastest on ferric ammonium citrate, while slower growth was observed on ferric oxide, and the slowest growth occurred on goethite (Fig. 1). In all three media, the growth rate of the wild type exceeded the growth rate of the Δ*sll1694* deletion strain.

As vortexing is a very efficient method for removing pili from cells (as practiced for pili isolation), we suspected that pili may also be constantly sheared off in agitated liquid culture. To prevent the potential for shearing off of pilins and to allow for a persistent contact with iron oxide particles, we assessed growth of wild type and the Δ*sll1694* strain on agar plates.

**Figure 2:**
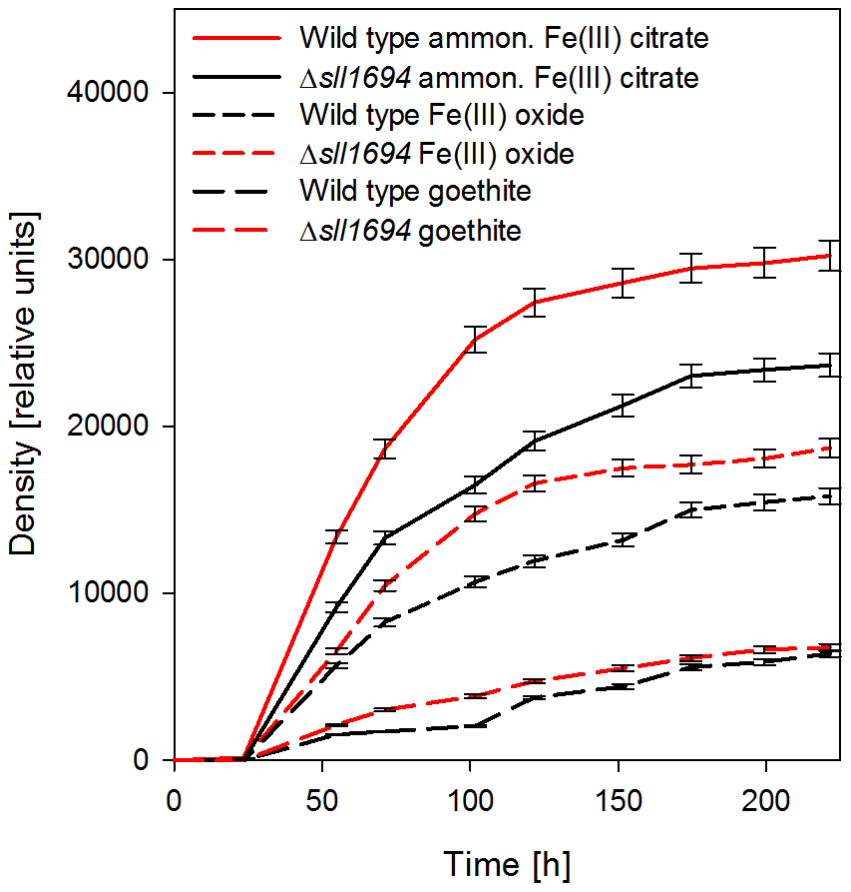
Agar-plate growth characteristics. Photoautotrophic growth characteristics of wild type and the Δ*sll1694* strain on BG-11-contatining agar plates with ammonium iron(III) citrate, iron(III) oxide, or goethite as the exclusive iron source. Trend shown is the average of three separate experiments. Error bars showing the standard error of the three experiments.

For wild-type cells grown on plates, ferric ammonium citrate, ferric oxide, and goethite showed the same propensity for supporting growth as observed in liquid medium; however, on plates, the differences in growth between wild type and the Δ*sll1694* strain were more exaggerated than in liquid culture (Fig. 2). While ferric citrate supported growth of Δ*sll1694* cells, very slow growth was observed on ferric oxide and goethite.

**Figure 3:**
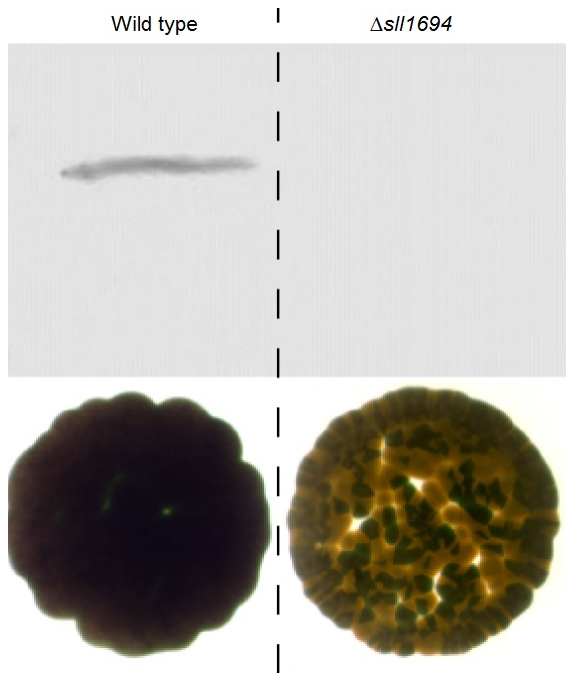
Agar-plate growth phenotype. Images of wild type (left) and Δ*sll1694* strain (right) on petri dishes containing agar-solidified BG-11 medium with ammonium iron(III) citrate substituted by goethite. The extracellular protein harvested from wild type and Δ*sll1694* strains show the PilA protein (encoded by *sll1694*) in the wild type, but not the Δ*sll1694*.

Agar-grown Δ*sll1694* cells exhibited a dramatic change in coloration on all iron sources, including ferric ammonium citrate (Fig. 3), while no obvious difference in coloration between wild type and the Δ*sll1694* strain were observed in liquid culture. The speckled phenotype of the Δ*sll1694* strain grown on solid media may be indicative of the statistical nature of having access to iron oxide particles.

### Whole cell absorption spectra

Absorption spectra of whole cells can provide information on the organization of the photosynthetic machinery of cyanobacteria. In *Synechocystis* sp. PCC 6803, absorption features can be readily assigned to chlorophyll *a*, carotenoids and phycobilins. In non-stress conditions, chlorophyll *a* is mainly associated with PSI and photosystem II (PSII) and exhibits absorption maxima around 435 nm (Soret band) and 680 nm. The main light-harvesting system of PSII is made up of phycobilisomes, which contain phycocyanin (absorption maxima at 570 nm and 620 nm) and to a lesser extent allophycocyanin (maxima at 650 nm), resulting in a composite absorption peak around 625 nm. It is possible to assess the relative chlorophyll / phycobilin ratio by evaluating the ratio of the 625 nm to 680 nm absorption peaks.

*Synechocystis* sp. PCC 6803 cells possess four main carotenoids [33] (β-carotene, zeaxanthin, myxoxanthophyll and echineone) that absorb light efficiently between 400 and 500 nm. The light absorption of the carotenoids therefore overlaps with the light absorption of chlorophyll *a*. An approximation of the amount of carotenoids within a cell can be obtained by assessing the peak ratio of chlorophylls at 435 nm and 680 nm. When Abs 435 nm / Abs 680 nm is small then a relatively small number of carotenoids are present. To unify replicated absorption spectra the statistical measurement of the standard error between the replicates was measured.

### Carotenoids

**Figure 4:**
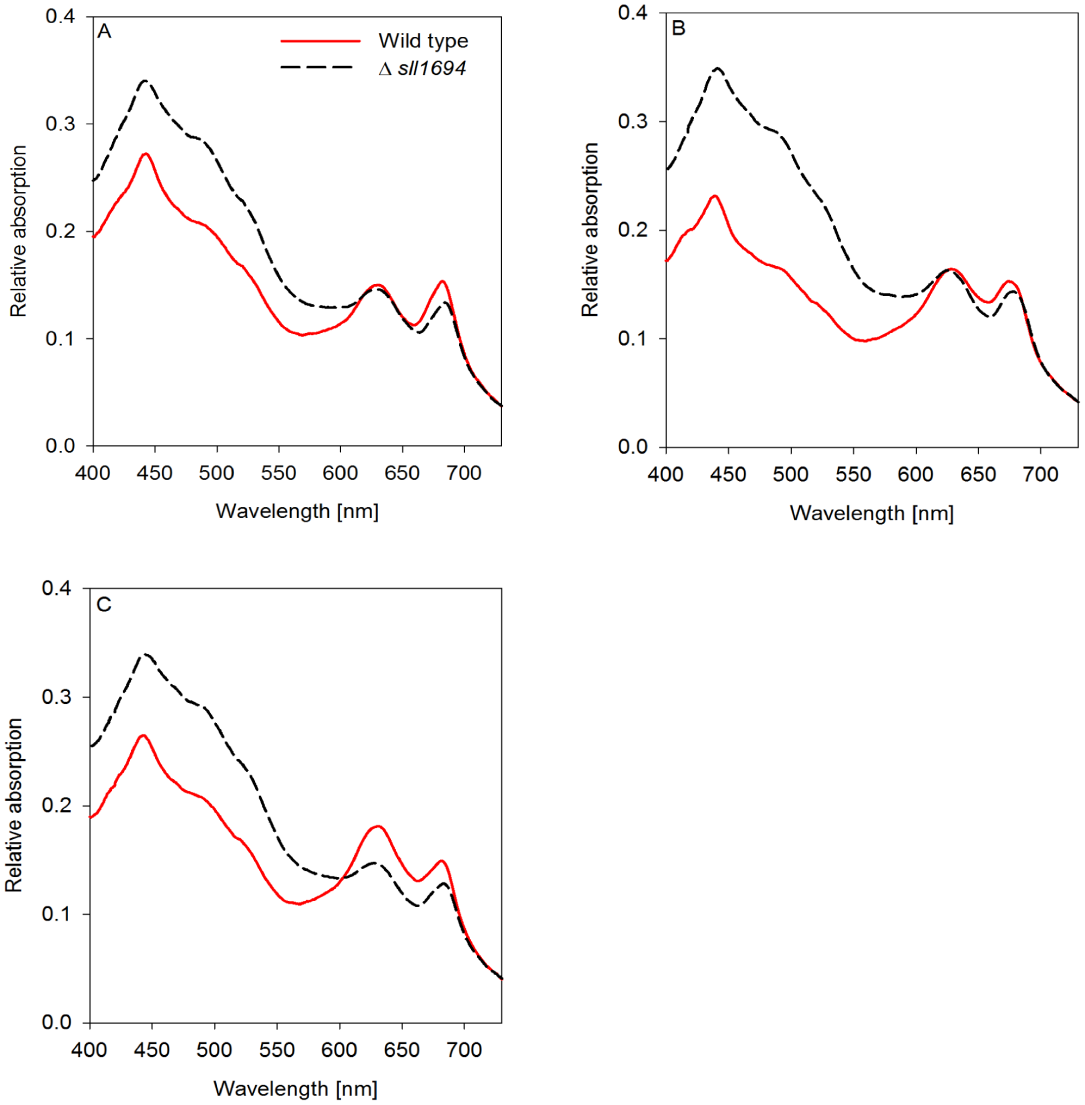
Absorption spectra of a photoautotrophically grown liquid culture. Both wild type and Δ*sll1694* strains grown in BG-11 with ammonium iron(III) citrate (A), iron(III) oxide (B), or goethite (C) as the exclusive iron source. Samples were standardized to an OD_800_ of 0.3. Traces were baseline subtracted at 800 nm after acquisition. One data set is shown, which is representative of three separate experiments. The average standard error between triplicates was calculated. Wild type SE: ±3.0×10^-3^ (A), ±1.5×10^−3^ (B), ±2.9×10^−3^ (C); Δ*sll1694* SE: ±3.3×10^−3^ (A), ±3.9×10^−3^ (B), ±3.7×10^−3^ (C). (For non-baseline subtracted absorption spectra see Figure S4 in File S1).

The absorption ratio at 435 nm and 680 nm was used to assess the presence of carotenoids in relation to chlorophyll *a* in wild type and the Δ*sll1694* cells grown on agar plates and in liquid, which were supplied with different iron sources. Wild type and the Δ*sll1694* strain have a similar carotenoid to chlorophyll ratio when grown in liquid with ferric ammonium citrate as the iron source (Fig. 4A). An increase in the carotenoid to chlorophyll ratio can be deduced in the Δ*sll1694* mutant compared to the wild type when either iron oxide or goethite was the iron source in liquid grown cultures (Fig. 4B, C).

**Figure 5:**
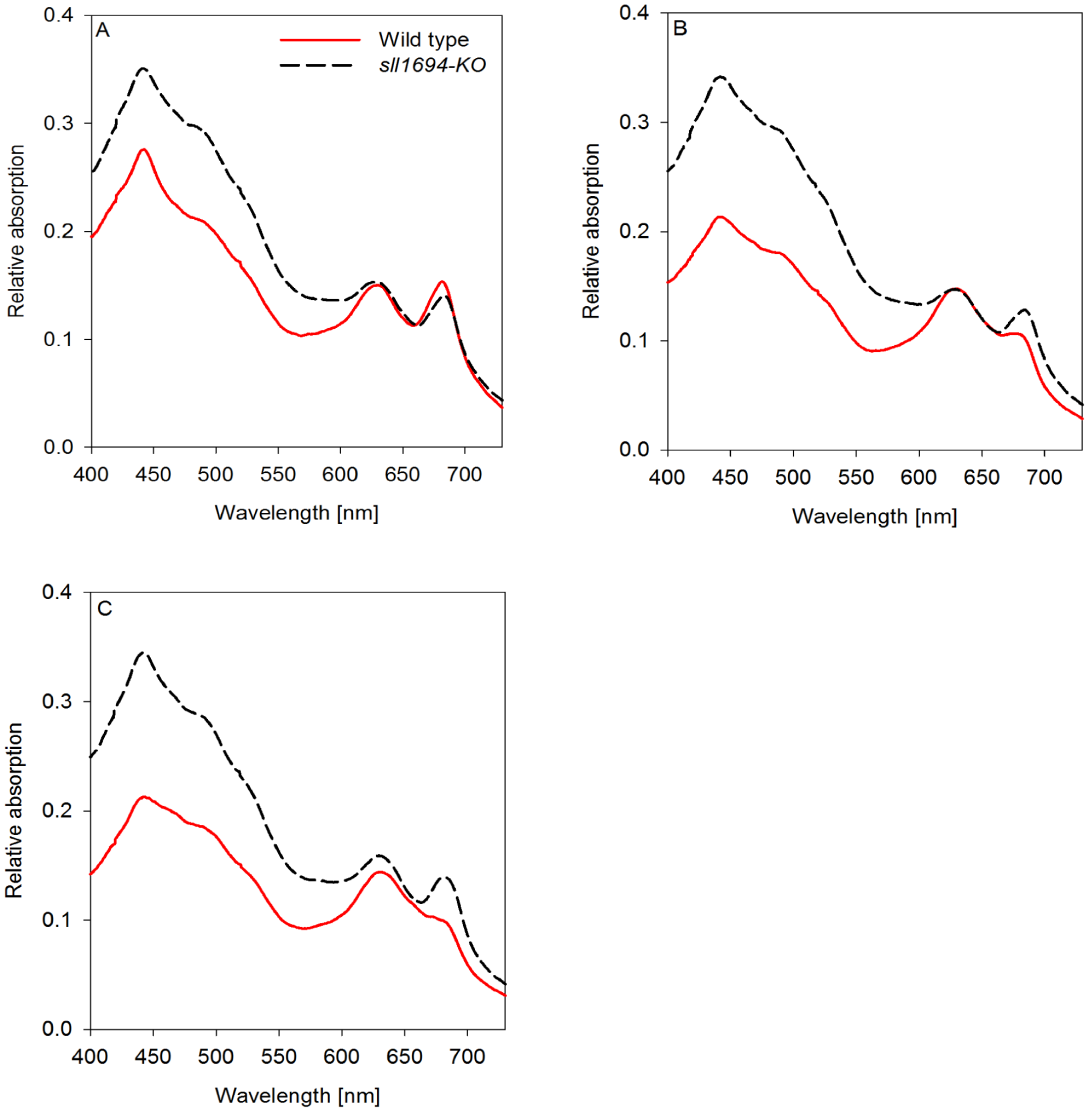
Absorption spectra of photoautotrophically grown plate cultures. Both wild type and Δ*sll1694* cells grown on agar-solidified BG-11 with ammonium iron(III) citrate (A), iron(III) oxide (B), or goethite (C) as the exclusive iron source. Samples were standardized to an OD_800_ of 0.3. Traces were baseline subtracted at 800 nm after acquisition. One data set is shown, which is representative of three separate experiments. The average standard error between triplicates was calculated. Wild type SE: ±1.8×10^−3^ (A), ±1.3×10^−3^ (B), ±3.9×10^−3^ (C); Δ*sll1694* SE: ±4.0×10^−3^ (A), ±4.3×10^−3^ (B), ±3.0×10^−3^ (C). For non-baseline subtracted absorption spectra for plate grown cultures see Figure S5 in File S1).

When grown on agar in the presence of ferric ammonium citrate, a similar carotenoid to chlorophyll ratio was present in the Δ*sll1694* strain and wild type (Fig 5A). In plate-grown cultures, the chlorophyll to carotenoid ratio was increased more in wild type than in the Δ*sll1694* strain grown on iron oxide and goethite (Fig 5B, C).

### Chlorophyll

The status of the photosynthetic machinery can be deduced from the amount of light-harvesting pigments that are present. In non-stress conditions, a relative high phycobilisome to chlorophyll ratio indicates active electron generation by PSII, while in stress conditions the number of phycobilisomes to chlorophyll is often reduced. The absorption at 625 nm and 680 nm can be used to assess changes in the phycobilisome to chlorophyll ratios in wild type and the Δ*sll1694* strain.

In liquid media, the Δ*sll1694* strain and wild type have similar chlorophyll to phycobilin ratios under two of the iron sources (ferric ammonium citrate, iron oxide) (Fig 4). When grown in liquid with goethite (Fig 4C), a small decrease in the chlorophyll to phycobilin ratio was observed in both wild type and Δ*sll1694* cells compared to cells grown on ferric ammonium citrate and ferric oxide.

On solid medium the Δ*sll1694* strain and wild type only have a similar chlorophyll to phycobilin ratio when grown on ferric ammonium citrate (Fig 5A). The wild type showed a substantial decrease in the chlorophyll to phycobilin ratio when grown on goethite and ferric oxide (Fig 5B & C).

### IsiA

In iron-limited conditions a shift in the red chlorophyll absorption peak to shorter wavelengths has previously been observed [34]. This shift has been attributed to the presence of IsiA [34], a chlorophyll-binding protein that is related to CP43. Unlike CP43, which is associated with PSII, IsiA has been shown to be primarily associated with PSI.

In liquid-grown wild-type cells and the Δ*sll1694* strain, a shift of the red chlorophyll peak to shorter wavelengths was observed when ferric ammonium citrate was replaced by ferric oxide (1–2 nm) and goethite (∼5–7 nm). The same trends were observed when wild type and the Δ*sll1694* strain were grown on plates with different iron sources (see Table 1).

**Table 1:** Absorption maxima of the red chlorophyll peak in wild type and Δ*sll1694* strain grown with different iron sources in liquid medium and on agar plates.

	wild type		Δ*sll1694*	
	liquid	plate	liquid	plate
**Iron(III) citrate**	682 nm	682 nm	685 nm	685 nm
**Iron oxide**	681 nm	678 nm	684 nm	684 nm
**Goethite**	675 nm	675 nm	680 nm	682 nm

### 77 K Fluorescence

The fluorescence spectra of whole cells at 77 K have been used to characterize the photosynthetic machinery of cyanobacteria [35]. Several fluorescence features can be assigned to specific photosynthetic complexes in *Synechocystis* sp. PCC 6803. The core light-harvesting complexes of PSII have characteristic fluorescence emission maxima at 685 nm (CP43) and 695 nm (CP47). Additionally, fluorescence emission at 685 nm has been found to arise from the terminal phycobilisome emitter, called Lcm [36], and IsiA [37]. PSI exhibits an emission maximum at 725 nm. In cyanobacteria, excitation with light at 440 nm preferentially excites chlorophyll *a* molecules that are part of PSI, PSII and IsiA, but not phycobilisomes (the main light-harvesting system associated with PSII of cyanobacteria). Validity of 77 K spectra is analyzed using the statistical measurement of the SE between replicates.

### Spectral characteristics of cells grown in liquid and on agar

**Figure 6:**
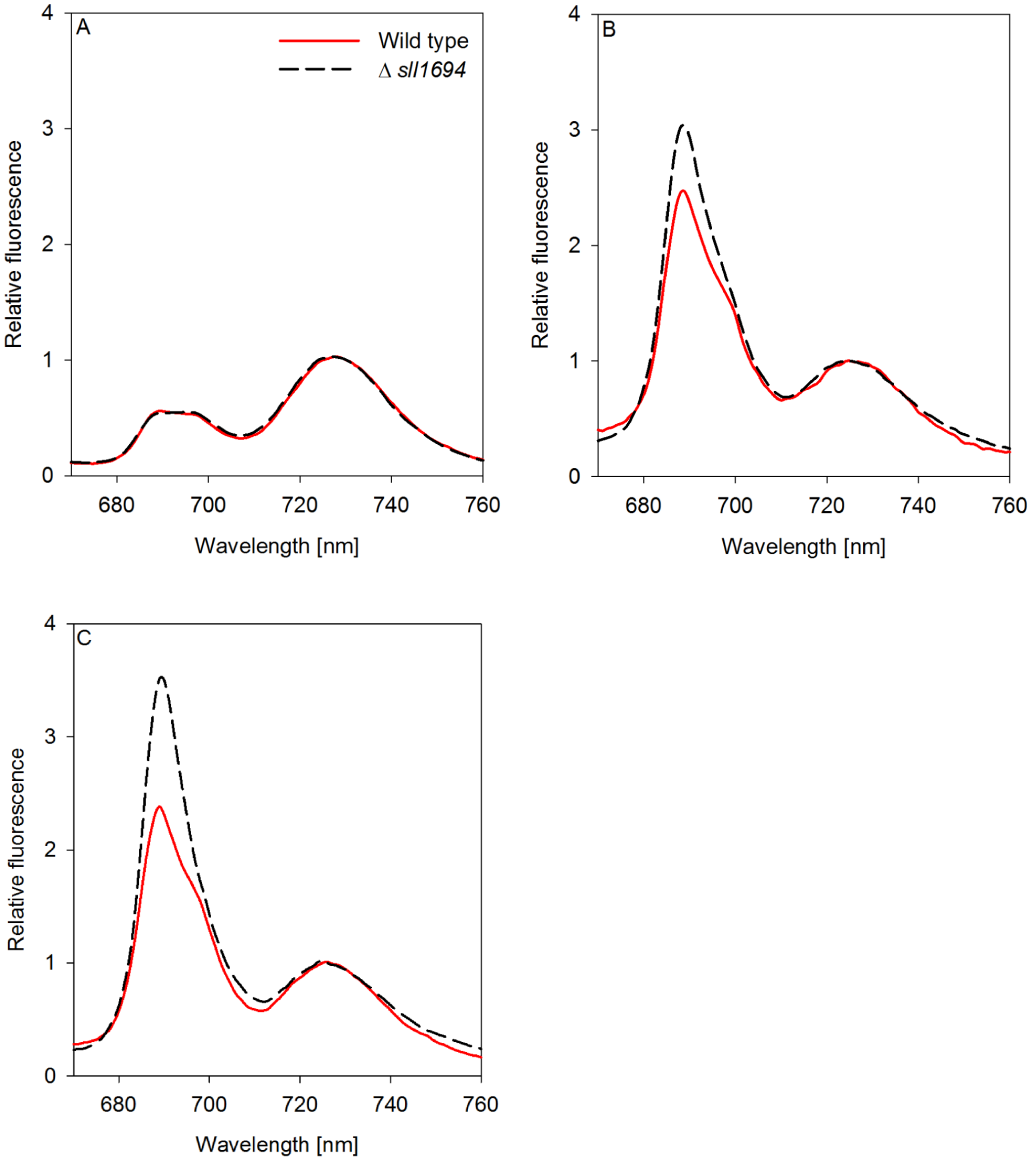
77 K fluorescence emission spectra of photoautotrophically grown liquid cultures excited by 440 nm light. Both wild type and Δ*sll1694* cells were grown in BG-11 with ammonium iron(III) citrate (A), iron(III) oxide (B), or goethite (C) as the exclusive iron source. Samples were excited by a 440 nm light source. Traces were normalized to the PS I peak at 725 nm. Data shown is indicative of three separate experiments. The average standard error between triplicates was calculated. Wild type SE: ±1.3×10^−2^ (A), ±2.3×10^−2^ (B), ±1.8×10^−2^ (C); Δ*sll1694* SE: ±1.7×10^−2^ (A), ±1.2×10^−2^ (B), ±1.0×10^−2^ (C).

Cells grown in liquid medium with different iron sources were investigated using fluorescence emission spectra at 77 K (Fig. 6 A). When iron was supplied as ferric ammonium citrate, the wild type and the Δ*sll1694* strain show similar chlorophyll *a* emission spectra that conform with 77 K emission spectra generally reported for wild type grown in liquid. Interestingly, an increase in the 685 nm fluorescence emission in wild type and the Δ*sll1694* strain was observed when iron oxide or goethite were supplied (Fig. 6 B, C) in liquid culture.

**Figure 7:**
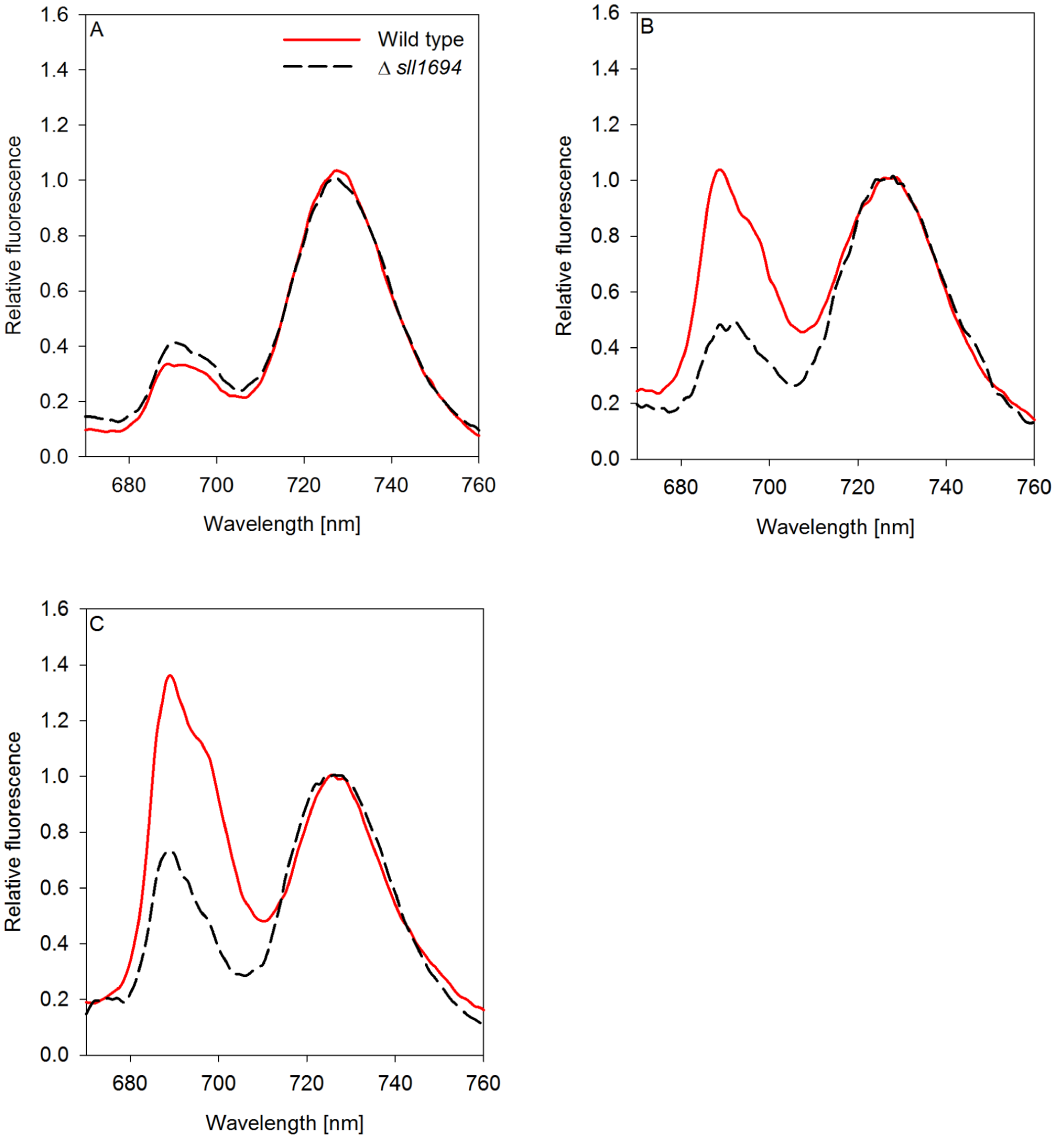
77 K fluorescence emission spectra of photoautotrophically grown plate cultures excited by 440 nm light. Both wild type and Δ*sll1694* cells grown on BG-11 plates with ammonium iron(III) citrate (A), iron(III) oxide (B), or goethite (C) as the exclusive iron source. Samples were excited by a 440 nm light source. Traces were normalized to the PS I peak at 725 nm. One data set is shown, which is representative of three separate experiments. The average standard error between triplicates was calculated. Wild type SE: ±2.7×10^−2^ (A), ±3.2×10^−2^ (B), ±3.5×10^−2^ (C); Δ*sll1694* SE: ±4.4×10^−2^ (A), ±4.2×10^−2^ (B), ±3.1×10^−2^ (C).

Under some conditions, the fluorescence emission spectra at 77 K of cells grown on agar (Fig. 7) contrast substantially to the spectra of the liquid-grown cells. In both, wild type and Δ*sll1694* strains, the fluorescence emission spectra from plate-gown cells looked similar to cells grown in liquid. In contrast, when goethite and iron oxide were supplied, the 685 nm and 695 nm peaks increased in the wild type, while a smaller increase was observed in the Δ*sll1694* strain.

To distinguish the contribution of IsiA and PSII to the 77 K fluorescence emission spectra, analysis of the ratio of the emissions specific for these complexes is required. The emission at 695 nm in relation to the emission peak at 685 nm indicates the presence of PSII and IsiA, respectively. This analysis revealed that slightly more IsiA is present in the Δ*sll1694* strain compared to the wild type in liquid-grown cells supplied with iron oxide and goethite (Fig. 6 B, C). Conversely, more IsiA is present in the wild type than in the Δ*sll1694* strain when grown on plates with iron oxide and goethite (Fig. 7 B, C).

The PSI and PSII ratio is another characteristic that can be evaluated by interpreting 77 K fluorescence emission spectra. Here the fluorescence emission at 695 nm indicates the presence of PSII, while the emission centered at 725 nm represents the presence of PSI. The spectra in Figs. 6 and 7 are normalized to the PSI emission peak at 725 nm. After taking into account of the contribution of IsiA to the spectra at 685 and 695 nm, it can be concluded that the ratio of PSII to PSI in liquid-grown cells is much higher than in the agar-grown cells for both wild type and the Δ*sll1694* strain on iron oxide and goethite. On agar, the wild type maintains a higher PSII to PSI ratio on iron oxide and goethite compared to the Δ*sll1694* strain.

## Discussion

Our study investigated the role of PilA1 in the utilization of oxidized (ferric) iron sources. Iron is a crucial cofactor of many protein complexes that mediate photosynthetic electron transport in cyanobacteria. Moreover, iron is a growth-limiting factor in many environments. Utilization of iron oxides through electron donation to the iron may therefore be an important strategy for survival in otherwise iron-limited environments.

Electron donation to iron oxides as a mechanism for utilizing otherwise inaccessible sources of iron has recently been suggested to occur in cyanobacteria [14]. Extracellular or periplasmic reduction of ferric iron is thought to be followed by transport of the soluble ferrous iron through the cytoplasmic membrane into the cell. In the proposed scheme, a ferri-siderophore is thought to be disassembled via reduction outside of the plasma membrane. This disassembly is followed by the uptake of the iron through the cytoplasmic membrane [14]. The reduction of iron oxides also occurs in non-photosynthetic soil bacteria [9]. Several mechanisms have been proposed for this respiratory reduction including the use of electrically conductive pili that link cellular electron transport to extracellular electron acceptors [9]. Based on this information, we decided to investigate if PilA1, the main constituent of pilins [28], [29], [30], [31], [38], [39], has a role in iron acquisition in cyanobacteria.

### Growth experiments

In our study, the lack of PilA1 has direct phenotypic consequences when cells are grown on plates, even when the biologically accessible iron source, ferric ammonium citrate, is present. Interestingly, the *pilA1*-deficient mutant strains exhibit a mosaic of pigmentation when grown on plates on ferric ammonium citrate. This phenotype may indicate that direct contact with iron oxide particles is required for iron utilization as proposed by Kranzler and colleagues [14]. Despite this, the difference between the Δ*sll1694* mutant and wild type indicates that another mechanism of iron reduction exists, as no change in pigmentation is observed in the wild type. In the wild type, extracellular PilA1 may enable the activation and utilization of oxidized iron sources while this is only possible when direct contact is made in the Δ*sll1694* mutant. Spectroscopic data reveals that this change in pigmentation is most likely the consequence of a decrease in phycobilisomes in the Δ*sll1694* strain.

Wild type and the Δ*sll1694* strain are able to grow on iron oxide and goethite in liquid cultures, although it is unlikely that they can make sustained contact with iron oxide mineral particles. Moreover, although the initial growth of wild type and Δ*sll1694* cells appears similar in liquid on iron oxide and goethite, the wild type shows more growth after several days. As pili may constantly be sheared off in liquid the ability to grow on iron in liquid culture indicates that in addition to the “cell-contact” iron utilization mechanism, an additional ferric iron uptake mechanism is also present, that cannot be engaged on plates.

**Figure 8:**
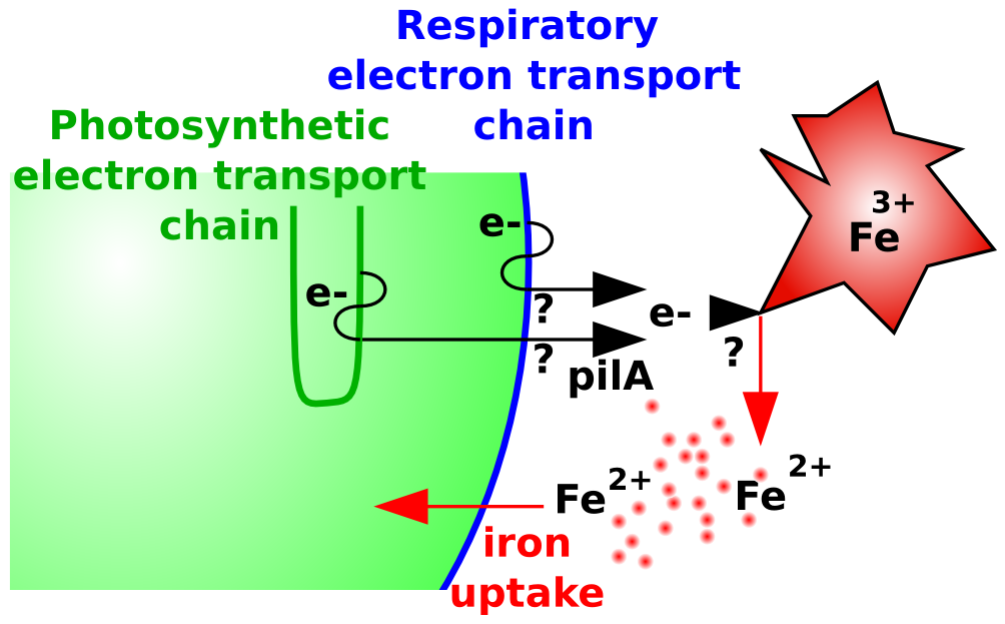
Schematic of electron transport to extra-cellular particles containing ferric iron. Ferric iron (Fe^3+^) is converted to ferrous iron (Fe^2+^) that can be taken up by a cyanobacterial cell. Electron transport indicated in black, thylakoid membranes indicated in green, respiratory membranes indicated in blue.

Our growth experiments on agar plates show that iron oxide and goethite can be used by the *Synechocystis* sp. PCC 6803 wild-type strain, while a PilA1-deficient strain struggles to utilize these sources of iron. As the PilA1-deficient mutant grows slower than the wild type on oxidized iron minerals, it seems likely that pili play an important role in accessing this iron on agar plates. The ability to move that is connected to the presence of pili in some *Synechocystis* sp. PCC 6803 strains is lost in the strain we used for our experiments. Resequencing of this strain [40] revealed a frameshift mutation in slr0162, whose gene product, PilC, is required for twitching motility. Therefore locomotion by the wild-type cells is not the reason for the observed phenotype. The PilA1 protein may be essential in the transport of electrons from the electron transport chain to iron oxides, and the consequent reduction of ferric iron into soluble ferrous iron (Fig. 8); the electron carriers that mediate this reduction of iron sources remain to be defined.

### IsiA

IsiA is induced under iron-limited conditions and therefore the presence of IsiA may be a good indicator for the availability of iron [41]; however, although IsiA has originally been discovered during iron stress, recent experiments also indicate that IsiA is induced as a response to oxidative stress [42].

Spectroscopic data at room temperature and fluorescence emission spectra at 77 K of wild type and the Δ*sll1694* strains indicate the presence of IsiA when cells are grown on oxidized iron minerals in liquid; however, the shift to shorter wavelengths associated with the presence of IsiA is more exaggerated in the wild type than in Δ*sll1694* strain. It is furthermore notable that the Δ*sll1694* strain possesses an *in vivo* room temperature light absorption spectrum that is further red-shifted than the wild type when grown under every iron source tested, including the bio-available ferric ammonium citrate. Possible reasons for this red-shift are an increased amount of IsiA in wild type and/or an increased amount of PSI in the Δ*sll1694* strain. Indeed, 77 K fluorescence emission data indicate that this combination of factors is present in agar-grown cells. Moreover, 77 K fluorescence spectra also indicate that the origin of this shift is not necessarily due to changes in the amount of IsiA and PSI, as the Δ*sll1694* strain and wild type possesses almost identical 77 K fluorescence emission spectra when grown on ferric ammonium citrate on agar plates and in liquid.

We have therefore to accept – at first sight – a contradicting situation in regards to iron utilization and presence of IsiA: growth tests indicate that PilA1-deficient cells are not able to utilize oxidized iron minerals as efficiently as wild type, but in response to iron limitation, PilA1-deficient cells only produce elevated amounts of the stress-response protein IsiA in liquid culture, but not on plate. We think this behavior can be explained by the physiology of plate-grown colonies, and by attributing the production of IsiA to the presence of oxidative stress and not the absence of iron.

The physiological response of *Synechocystis* sp. PCC 6803 wild type and the Δ*sll1694* strain to growth on iron oxide minerals in liquid is somewhat surprising as the absorption spectra indicate that phycobilisomes are abundant and 77 K fluorescence emission spectra indicate the presence of IsiA in coordination with a high PSII to PSI ratio. This contrasts with the general observation of liquid-grown, iron-limited cyanobacteria cultures, where an increase in the amount of IsiA and a decrease in the amount of phycobilisomes and PSII are observed [43]. One interpretation of these data is that the wild-type cells experience some stress but are not iron-limited. This stress may originate from a higher oxygen concentration present within dense cyanobacterial colonies, e.g. bacterial mats [44].

### Possible regulatory network

Recent transcriptome profiling has uncovered a wealth of expressed antisense and other noncoding RNAs in *Synechocystis* sp. PCC 6803 [45]. Within the *pilA12* operon (*sll1693, sll1694, sll1695* and *sll1696*), two asRNA molecules (*sll1694*-as and *sll1695*-as), along with three other non-coding RNA features have been detected [45]. The deletion and over-expression of these antisense RNA will aide in determining their function, including the regulation of the *isiA* operon.

A study that investigated the transcriptional response of an *isiA* deletion strain provides evidence that pilins are a component in a complex stress response network. An *isiA* deletion mutant exhibited an increase in the mRNA of the pilin-containing *sll1693-sll1696* operon and two other pilin genes (*slr1456* and *slr0079*) compared to wild type during standard growth conditions [27]. The *isiA-isiB* operon in turn also contains a regulatory mRNA (*isiR*) that would have been impaired by deleting *isiA* in the aforementioned study. The presence of regulatory RNAs in the pilin-containing operon *sll1694-sll1695* and the *isiA-isiB* operon, in combination with the phenotypic coupling of these operons observed by Singh and colleagues [27] as well as our study, suggest that a complex regulatory framework coordinates PilA1 and IsiA expression. This assessment is in line with our observations, as iron metabolism and oxidative stress often interface with one another, and therefore may require complex regulation.

In our Δ*sll1694* deletion strain, the pilin *sll1695* (PilA2) and a protein with an unknown function (*sll1696*) are likely not to be expressed due to the disruption of the operon. Inactivating each gene in this operon, while keeping the other genes intact will be crucial to pinpoint the component that is critical for the observed phenotype of the mutant we generated. This approach will also be useful to assess the function of regulatory elements that are present in the *sll1693-sll1696* operon.

## Conclusions

Our study provides evidence that *Synechocystis* sp. PCC 6803 is well adapted to growing on a solid medium. *Synechocystis* and *Synechococcus* species are part of microbial mats and therefore growing in a high-oxygen environment should be part of their physiological repertoire. In the case of *Synechocystis* sp. PCC 6803, the ability to form fast-growing colonies on plates is likely dependent on tolerating high oxygen concentrations.

The presented work shows that the absence of PilA1 in the cyanobacterium *Synechocystis* sp. PCC 6803 impairs growth on iron oxide minerals. This is the first report that connects pilins and by extension pili with iron oxide utilization in cyanobacteria. Presently, we cannot clearly distinguish if the observed phenotypic consequences are solely caused by the inability to utilize oxidized iron sources, or are also a consequence that arises due to the disruption of a regulatory network, or a combination of both. Nonetheless, if other bacteria can utilize pili to donate electrons to iron oxides, it would appear unlikely that cyanobacteria would not have developed this capability. The ancestors of modern cyanobacteria were surely the first to experience the consequences of elevated oxygen concentrations. It would therefore seem not surprising that cyanobacteria have developed or adapted a tool that allows them to unlock the non-bioavailable metal oxides their oxygen produces.

## Materials and Methods

### Growth and maintenance of stock cultures

Cells were maintained on BG-11 agar plates containing 5 mM glucose, 20 µM atrazine and appropriate antibiotics where applicable [46]. Liquid cultures were established in 300 mL Erlenmeyer flasks that have been specifically modified as described by Eaton-Rye [46]. Cultures containing BG-11, 5 mM glucose and appropriate antibiotics were grown mixotrophically. Both plates and liquid cultures were grown under constant illumination (30 µE.m^−2^.s^−1^), at 30°C. Liquid cultures were provided with filtered aeration via small aquarium pumps. The air was additionally bubbled through ddH_2_O to prevent dehydration of cell cultures.

### Alternative iron sources

BG-11 media without ammonium iron(III) citrate was supplemented with either 1.84 µg.mL^−1^ (23 µM Fe) of iron(III) oxide, or 2.04 µg.mL^−1^ (23 µM Fe) of goethite. BG-11.

### Experimental liquid-media growth curve

Liquid cultures of *Synechocystis* sp. PCC 6803 were grown in the presence of 5 mM glucose to an OD of 1.0. Cells were centrifuged at 2760 g for 10 min at 25°C and washed twice in BG-11. A flask containing 150 mL BG-11 (without glucose or atrazine) was inoculated with washed cells to give a starting OD of 0.05. Cultures were maintained under constant temperature (30°C), illumination (30 µE.m^−2^.s^−1^) and aeration. The OD of each culture was recorded every 12 h over 148 h period using a custom built spectrophotometer [47]. To measure the OD accurately, a small sample of the culture was taken and diluted 5 – fold to yield the true culture OD.

### Experimental solid-media growth curve

Liquid cultures of *Synechocystis* sp. PCC 6803 were grown in the presence of 5 mM glucose to an OD of 1.0. Cells were centrifuged at 2760 g for 10 min at 25°C followed by two washing steps in sterile water and resuspended to an OD of 1.0. A small amount of this liquid culture was used to inoculate 2 mL of sterile water to an OD of 0.1. Serial dilutions of this culture were subsequently made to attain two further OD aliquots of 0.01 and 0.001. Two micro liters of each of the three culture samples were spotted onto BG-11 (without glucose or atrazine) agar growth plates with appropriate growth conditions (specific iron source). Cultures were maintained under constant temperature (30°C), illumination (30 µE.m^−2^.s^−1^) and aeration. Raw images of each culture were taken every 24 h over a 220 h period using a custom built plate imager. The integrated intensity of the cultures was compared to the agar background to give a relative growth parameter.

### Polymerase chain reaction

Primers for PCR analysis were ordered as needed in the 5′ to 3′ orientation with specific restriction enzyme cut sites upstream of the 5′ end (Table. S1 in File S1), through Sigma-Aldrich (Sigma-Aldrich, NSW, Australia).

The reactions were performed in 1x Phusion amplification buffer containing 0.4 mM of each dNTP (dATP, dCTP, dGTP and dTTP), 0.4 mM of each primer and 0.05 units/µL Phusion polymerase (Thermo Fisher Scientific, USA). PCR conditions were: (1) initial denaturing step at 98°C for 30 s; (2) 14 cycles of 98°C for 7 s, annealing at 62°C (−1°C per cycle) for 20 s, extension at 72°C for 30 s/kb; (3) 16 cycles of 98°C for 7 s, annealing at 62°C for 20 s, extension at 72°C for 30 s/kb; then (4) final extension at 72°C for 5 min. PCR products were cleaned using a QIAquick PCR purification kit (Qiagen, Duesseldorf, Germany).

### Separation of DNA samples by gel electrophoresis

DNA samples were separated by electrophoresis using 0.8% agarose gels in the presence of 10 mM NaOH and 73 mM boric acid at pH 8.0. The running condition was 150 V for 25 min at room temperature. Prior to loading, samples were mixed with 10× loading buffer (0.25% bromophenol blue, 0.25% xylene cyanol FF and 30% glycerol). DNA was visualized by exposure to a UV light after soaking the gel in ddH_2_O containing 1 mg/mL ethidium bromide for 10 min. Gel images were captured using a GelDoc (BioRad, USA).

### Plasmid construction

Ligation reactions were carried out at a vector to insert ratio of 3∶1 with ∼100 ng DNA in total. The reaction contained 50 mM Tris-HCl (pH7.5), 10 mM MgCl_2_, 10 mM dithiothreitol (DTT), 1 mM ATP, and 3 U of T4 DNA ligase (Roche, Mannheim, Germany). The reaction mixture was incubated at 22°C overnight.

### Transformation of *Synechocystis* sp. PCC 6803

Liquid cultures were grown in the presence of glucose to an OD of approximately 0.5. Cells were then centrifuged at 2760 *g* for 10 min and suspended in 0.5 mL BG-11 media to a final OD of 2.5 in a sterile glass tube. Approximately 5 µg of plasmid DNA was added to tubes and incubated at 30°C under 30 µE.m^−2^.s^−1^ of illumination for 6 h with gentle shaking at the 3 h mark. Negative controls with no DNA were also included. Samples were spread over sterile nitrocellulose paper on BG-11 plates supplemented with glucose and incubated for 12 h. The filters, with cells attached, were then transferred to plates containing glucose, atrazine and appropriate antibiotics. After two weeks, single colonies were picked and streaked out weekly for three weeks to ensure complete segregation. Colony PCR was used to verify complete segregation using the *sll1694* left flank forward (NcoI) primer and the *sll1694* right flank reverse (PstI) primer.

### Whole cell absorption spectra

Whole cell absorption spectra were measured using a Jasco V-550 spectrophotometer. Cells were suspended in BG-11 containing HEPES-NaOH, pH 7.5, to an OD of 0.3. Translucent cellotape was fixed on both sides of the cuvette holder during measurements. Spectra were baseline subtracted after acquisition, during the data analysis.

### 77 K fluorescence

Cells were diluted with BG-11 containing HEPES/NaOH, pH 7.5, to a chlorophyll concentration of 2 µg.mL^−1^ measured using a custom built fluorometer [48]. Cells were then transferred into glass tubes, frozen in liquid nitrogen and fluorescence emission assessed using a fluorescence spectrometer (Perkin-Elmer, MPF-3L). The emission slit was set to 4 nm for each measurement. The excitation slit for samples was 12 nm and 10 nm for 440 nm and 580 nm, respectively. Traces were normalized to a PSI peak at 725 nm in the emission spectra.

### PilA protein harvest

Cells were grown in 200 mL of BG-11 to an OD of 0.8. They were then centrifuged at 2760 g for 8 min and the supernatant was discarded. The cell pellet was then re-suspended in 5 mL of BG-11. The cell suspension was subjected to 2 min of thorough vortexing followed by another centrifugation at 2760 g for 8 min. The supernatant was carefully removed without disrupting the cell pellet and concentrated using a Vivaspin 500 column with a 5 kDa molecular weight cut off (GE Healthcare, UK) to a final volume of 50 µL.

### Sodium dodecyl sulfate-polyacrylamide gel electrophoresis

In this study, 12% SDS-PAGE using a Tris-glycine buffer system was used. The running condition for Tris-glycine was 200 V for 55 min at room temperature. Directly after completion of electrophoresis, the gels were removed from the glass cassette and soaked in a gel-fixing solution (50% (v/v) ethanol and 10% (v/v) acetic acid in 18 MΩ resistant purified milli Q water) for 1 h. The gel was then incubated at room temperature overnight with slight agitation in gel-washing solution (50% (v/v) methanol and 10% (v/v) acetic acid in ddH_2_O). For visualization of protein bands the gel was covered with Coomassie stain (0.1% (w/v) Coomassie blue R350, 20% (v/v) methanol and 10% (v/v) acetic acid in 18 MΩ resistant purified milli Q water) for 3 to 4 h with slight agitation. Destain solution (50% (v/v) methanol and 10% (v/v) acetic acid in 18 MΩ resistant purified milli Q water) was then used several times to remove excess Coomassie stain. The gel was then stored indefinitely in a storage solution (5% (v/v) acetic acid in 18 MΩ resistant purified milli Q water.

### Mass spectrometry analysis

Samples were excised carefully from the SDS-PAGE gel and analyzed at the Centre for Protein Research at the University of Otago in order to determine identity of the protein bands.

## Supporting Information

File S1 Contains the following files: Figure S1. Genomic DNA sll1694-deletion through homologous recombination. Figure S2. Gel showing colony PCR products. Figure S3. SDS-PAGE Analysis of extracellular proteins. Figure S4. Absorption spectra of liquid grown culture. Figure S5. Absorption spectra of plate grown culture. Table S1. PCR primers including specific cut sites.
(DOC)
