# Supplementary material for: Functional Role of PilA in Iron Acquisition in the Cyanobacterium Synechocystis sp. PCC 6803
Source: PLoS One. 2014 Aug 26;9(8):e105761. doi: 10.1371/journal.pone.0105761 (PMC4144903; doi:10.1371/journal.pone.0105761)
Supplement: File S1 — Contains the following files: Figure S1. Genomic DNA sll1694-deletion through homologous recombination. Figure S2. Gel showing colony PCR products. Figure S3. SDS-PAGE Analysis of extracellular proteins. Figure S4. Absorption spectra of liquid grown culture. Figure S5. Absorption spectra of plate grown culture. Table S1. PCR primers including specific cut sites. (DOC) [file pone.0105761.s001.doc]

**Supporting material for:**

Functional role of PilA in iron acquisition in the cyanobacterium *Synechocystis* sp. PCC 6803

This document includes:

Supporting figures:

Supporting Figure 1. Genomic DNA *sll1694-*deletion through homologous recombination
Supporting Figure 2. Gel showing colony PCR products
Supporting Figure 3. SDS-PAGE Analysis of extracellular proteins
Supporting Figure 4. Absorption spectra of liquid grown culture
Supporting Figure 5. Absorption spectra of plate grown culture

Supporting table:
 Supporting Table 1. PCR primers including specific cut sites

**
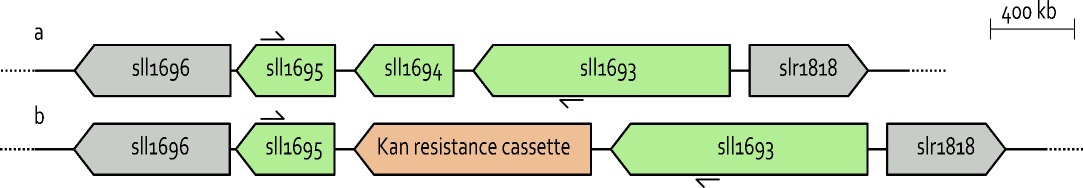
**

**Supporting Figure 1. Genomic DNA *sll1694-*deletion through homologous recombination.** (a) Wild-type chromosomal DNA from *Synechocystis* sp. PCC 6803 showing the position of *sll1694* relative to the flanking genes. (b) Chromosomal DNA after homologous recombination that replaces *sll1694* with the antibiotic-resistance cassette conferring resistance against kanamycin. Primers that were used to verify segregation (Fig. 2) are indicated.


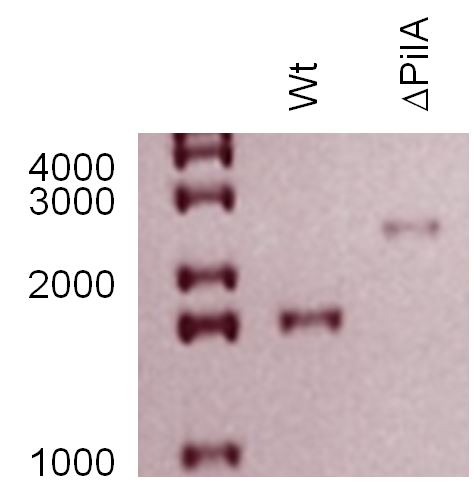


**Supporting Figure 2. Gel showing colony PCR products.** Colony PCR products of wild type, and the Δ*sll1694* mutant. The primers located on *sll1693* and *sll1695* fragment resulted in a 1.6 kb band for the wild-type DNA and a 2.6 kb band for the Δ*sll1694* mutant, respectively.


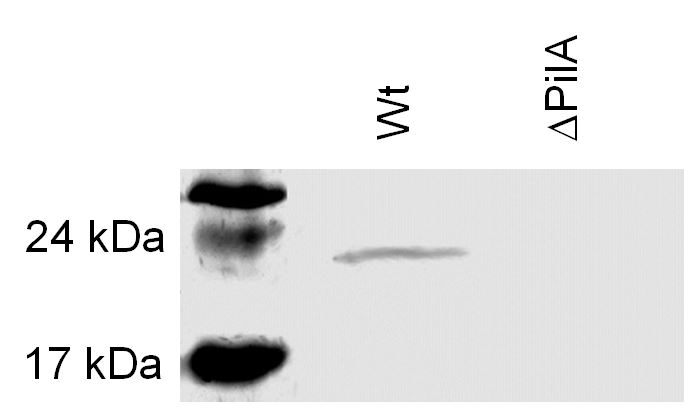


**Supporting Figure 3. SDS-PAGE analysis of extracellular proteins.** Extracellular protein harvested from wild type and Δ*pilA* strains were analyzed by SDS-PAGE. The protein band in the wild-type strain is that of the PilA protein as confirmed by MALDI TOF-TOF mass spectrometry.


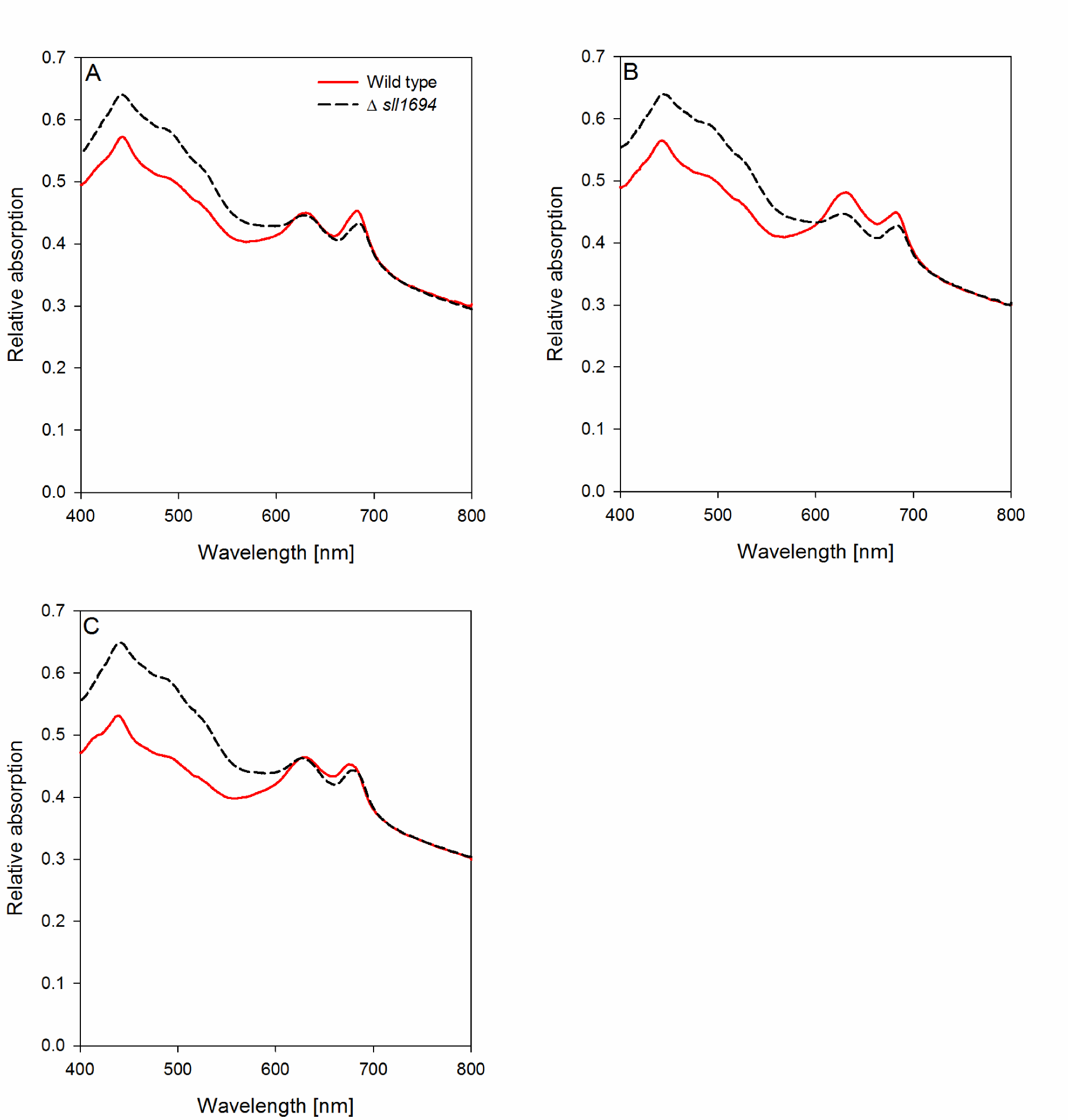


**Supporting Figure 4. Absorption spectra of liquid grown culture**. Both wild type and Δ*sll1694* cells grown in BG-11 with ammonium iron(III) citrate (A), iron(III) oxide (B), or goethite (C) as the exclusive iron source are shown. Samples were standardized to an OD_800_ of 0.3. Data shown are indicative of three separate experiments. The average standard error between triplicates was calculated. Wild type SE: ± 3.0 × 10^-3^ (A), ± 1.5 × 10^-3^ (B), ± 2.9 × 10^-3^ (C); Δ*sll1694* SE: ± 3.3 × 10^-3^ (A), ± 3.9 × 10^-3^ (B), ± 3.7 × 10^-3^ (C).


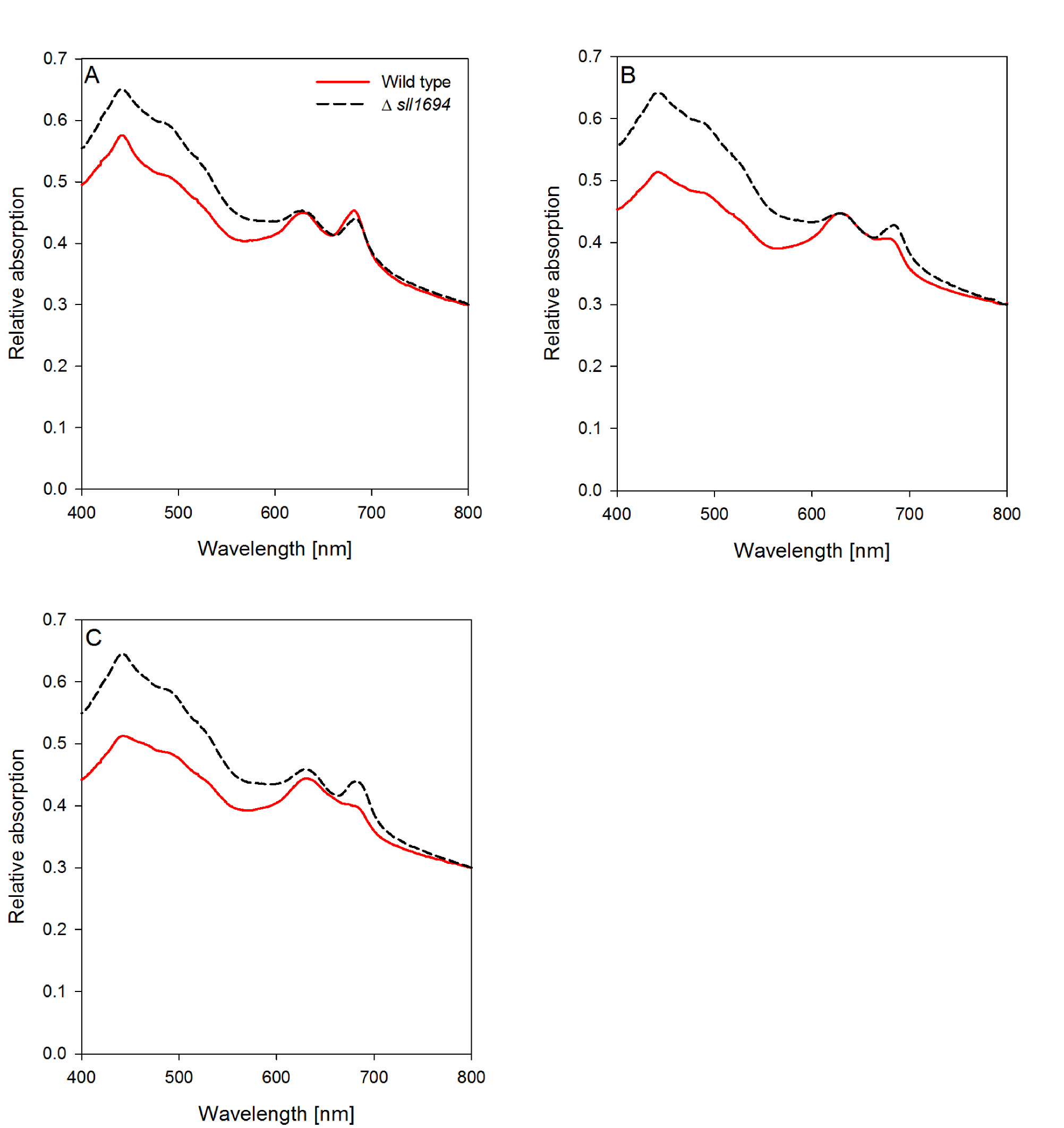


**Supporting Figure 5. Absorption spectra of plate grown culture**. Both wild type and Δ*sll1694* cells grown in BG-11 with ammonium iron(III) citrate (A), iron(III) oxide (B), or goethite (C) as the exclusive iron source are shown. Samples were standardized to an OD_800_ of 0.3. Data shown are indicative of three separate experiments. The average standard error between triplicates was calculated. Wild type SE: ± 1.8 × 10^-3^ (A), ± 1.3 × 10^-3^ (B), ± 3.9 × 10^-3^ (C); Δ*sll1694* SE: ± 4.0 × 10^-3^ (A), ± 4.3 × 10^-3^ (B), ± 3.0 × 10^-3^ (C).

**Supporting table**

Supporting Table 1. PCR primers including specific cut sites

| pUC19 forward (PstI) | ATCTGCAGGTTTTCCCAGTCACGAC |
| --- | --- |
| pUC19 reverse (NcoI) | ATCCATGGCAGGAAACAGCTATGAC |
| *sll1694* left flank forward (NcoI) | CTAGCCATGGTTGTCTTCTTCCTTCTGTAG |
| *sll1694* left flank reverse (SphI) | GTGGGCATGCCCAACCAGAATCTGCTTTG |
| Kanamycin-resistance cassette forward (SphI) | ATGCAGTCCAACAAAGCCACGTTGTGTC |
| Kanamycin-resistance cassette reverse (SacI) | ATGAGCTCGGAATAGCGCTGAGGTCTGC |
| *sll1694* right flank forward (SacI) | AAAAGAGCTCAATACCGTTTACACAGTC |
| *sll1694* right flank reverse (PstI) | GGTGCTGCAGTAATTAAATAGGACCC |
